# Klotho reduces the risk of osteoporosis in postmenopausal women: a cross-sectional study of the National Health and Nutrition Examination Survey (NHANES)

**DOI:** 10.1186/s12902-023-01380-9

**Published:** 2023-07-14

**Authors:** Jialin Jiang, Qinyu Liu, Yaqian Mao, Nengyin Wang, Wei Lin, Liantao Li, Jixing Liang, Gang Chen, Huibin Huang, Junping Wen

**Affiliations:** 1 Shengli Clinical Medical College of Fujian Medical University, Fuzhou, China; 2 Department of Endocrinology, Fujian Provincial Hospital, Fuzhou, China; 3 Department of Internal Medicine, Fujian Provincial Hospital Jinshan Branch, Fuzhou, China; 4 Fujian Provincial Key Laboratory of Medical Analysis, Fujian Academy of Medical, Fuzhou, China

**Keywords:** Postmenopausal osteoporosis, Klotho, NHANES

## Abstract

**Background:**

Osteoporosis (OP) is one of the diseases that endanger the health of the elderly population. Klotho protein is a hormone with anti-aging effects. A few studies have discussed the relationship between Klotho and OP. However, there is still a lack of research on larger populations. This study aims to evaluate the association between OP and Klotho in American postmenopausal women.

**Methods:**

This is a retrospective study. We searched the National Health and Nutrition Examination Survey (NHANES) database and collected data of 3 survey cycles, finally involving 871 postmenopausal women over 50 years old in the present study. All participants took dual-energy X-ray absorptiometry examination and serum Klotho testing at the time of investigation. After adjusting the possible confounding variables, a multivariate regression model was employed to estimate the relationship between OP and Klotho proteins. Besides, the *P* for trend and restricted cubic spline (RCS) were applied to examine the threshold effect and calculate the inflection point.

**Results:**

Factors influencing the occurrence of OP included age, ethnicity, body mass index and Klotho levels. Multivariate regression analysis indicated that the serum Klotho concentration was lower in OP patients than that in participants without OP (OR[log_2_Klotho] = 0.568, *P* = 0.027). The C-index of the prediction model built was 0.765, indicating good prediction performance. After adjusting the above-mentioned four variables, *P* values for trend showed significant differences between groups. RCSs revealed that when the Klotho concentration reached 824.09 pg/ml, the risk of OP decreased drastically.

**Conclusion:**

Based on the analysis of the data collected from the NHANES database, we propose a correlation between Klotho and postmenopausal OP. A higher serum Klotho level is related to a lower incidence of OP. The findings of the present study can provide guidance for research on diagnosis and risk assessment of OP.

**Supplementary Information:**

The online version contains supplementary material available at 10.1186/s12902-023-01380-9.

## Introduction

Osteoporosis (OP) is a condition that is detrimental to the health of perimenopausal and postmenopausal women. According to a cross section statistics [1], among the elders in the United States, 43.9% suffer from low bone mass, and 15.4% have OP, about four-fifths of whom are women. Thus, women are at high risk of OP. The bone loss during menopause is the main cause of OP in the elderly women. Women have smaller and thinner bones than men. As the ovary stops functioning and the circulating estrogen level drops sharply after menopause, the bone loss accelerates [2]. OP is a health hazard to elderly women. It can lead to fractures, body deformity, chronic pain, disability and loss of independence in elderly women, compromise their ability to walk, and ultimately impair their quality of life [3]. Osteoporotic hip fractures account for 5% of female deaths from all causes [4], severely harming women's life and health. Therefore, it is quite necessary to identify and detect OP early and adopt timely medical interventions.

Dual-energy X-ray absorptiometry (DXA) is currently the gold standard for OP diagnosis. DXA measures the bone mineral density (BMD) based on the difference in absorption of two different energy X-rays by the spine, neck of femur (or bones in other body parts), and their surrounding soft tissues. Generally, the mean and standard deviation (SD) of the BMDs of adult women who reach the peak bone mass are taken as the reference. If the BMD of a postmenopausal woman is less than 2.5SD of the BMDs in the same region, OP will occur [5]. However, DXA scanning is not recommended for population screening in most countries due to its high cost and essential needs for professional equipment which is awkward to carry and well-trained operators. In addition, in some countries and regions with a low economic development level, to screen for and detect OP is even harder. Therefore, it is extremely important to use relevant risk factors to identify OP early in clinical practice.

Nowadays, most countries screen for OP using an opportunistic case-finding strategy [6]. A series of OP scoring tools have been developed, including Osteoporosis Index of Risk (OSIRIS) [7], Simple Calculated Osteoporosis Risk Estimation (SCORE) [8], Osteoporosis Self-assessment Tool (OST) [9], Osteoporosis Risk Assessment Instrument (ORAI) [10], etc. The Frame Risk Assessment Tool (FRAX) [11] is a widely-used prediction tool released in 2008. It provides a country-specific algorithm for the estimation of an individual’s 10-year probability of incurring a hip joint fracture or a severe osteoporotic fracture. The model has been applied in 63 countries, covering 79% of the global population. The FRAX predicts the risk of OP in an individual by analyzing risk factors such as age, gender, weight, height, smoking history, and alcohol use history. However, with the deepening of research in the field of OP, an increasing number of factors influencing OP occurrence are being detected, and many factors entail correction according to regional and ethnic differences. Therefore, constant exploration for new risk factors is a necessity for achieving more accurate OP screening results.

Klotho is an anti-aging protein discovered by Kuro-o et al. [12] in 1997. Klotho was identified as a family of related proteins, with α-, β-, and γ- Klotho isoforms. α-Klotho is expressed mainly on the cell surface membrane of proximal and distal renal tubules [13] as well as in the choroid plexus of the brain [14]. Structurally, it is a transmembrane protein [15]. Its extracellular domain can be cleaved by protease to release soluble forms of Klotho [16]. Soluble Klotho (s-Klotho) is the main functional form in the circulation [17], which can be detected in blood, urine and cerebrospinal fluid samples. In fact, only α-Klotho has been shown to be released from the cell into the extracellular fluid, and there are no data on soluble forms β-Klotho and γ-Klotho. It can be confirmed that Klotho detected in the plasma is basically equivalent to α-Klotho [18].

There are several studies on Klotho. In a study, OP sheep models [19] were constructed through ovariectomy and glucocorticoid treatment, and kidney samples were collected from the sheep. The analysis results showed that the expression of Klotho in the ovariectomized sheep that took glucocorticoids for 2 months was higher than that in other groups. Shimada T et al. [20] found that Klotho-deficient mice showed the same aging phenotype as fibroblast growth factor-23 (FGF-23) null mice. Subsequent studies confirmed that Klotho was functionally a co-receptor of FGF-23 receptors [21, 22]. FGF-23 is a bone-derived hormone that can promote phosphate excretion and negatively regulate the level of VitD [23]. FGF23/Klotho pathway is of physiological significance in maintaining normal bone metabolism, and abnormal levels of FGF23 will bring about metabolic bone disease. The variation of Klotho is related to the BMD of the population [24–26]. Kužmová Z et al. [27] reported that Klotho was positively correlated with the trabecular bone score (TBS) in populations with chronic kidney disease (CKD), and negatively correlated with fracture risk. According to the study results of Baldan A et al. [28], a decreased Klotho concentration was associated with a higher probability of suffering from fragility fractures in β-Thalassemia patients. However, in the study of De-la–O A et al. [29], a negative correlation between BMD and Klotho levels was observed in middle-aged sedentary people. Some previous studies have explored the relationship between Klotho and OP, but these studies have a small sample size or involve populations with specific diseases. Therefore, the present study aims to investigate the relationship between Klotho and OP through a study of large representative populations.

## Materials and methods

### Database

The National Health and Nutrition Examination Survey (NHANES) is a project of the U.S. National Health Center. Focusing on health examination and the healthy diet in the United States, the NHANES collected comprehensive data on the diet, nutritional health status and chronic diseases. Beginning in 1999, the survey has been conducted continuously to collect data, and new data are released in a 2-year cycle, with each cycle involving approximately 10,000 participants. The NHANES captures nationally representative data of American citizens based on a complex stratified sampling survey design and population-specific sample weights. Briefly, a series of sample family interviews and standardized physical examinations were conducted in designated mobile examination centers (MECs) across the country. In the present study, we extracted the data from the NHANES database before August 1st, 2022. The survey data were divided into five categories: Demographic data, dietary data, examination data, laboratory data and questionnaire data. All NHANES-based research has been approved by the National Center for Health Statistics (NCHS) Research Ethics Review Board, and all participants have provided signed informed permission [30]. Ethical approval and more detailed information are available on the website of the Ethics Review Board of the National Center for Health Statistics (https://www.cdc.gov/nchs/nhanes/irba98.htm) [31].

### Study population and study design

We extracted and analyzed three two-year cycles (2007–2010 and 2013–2014) of published data. The present study initially enrolled 15537 female participants who were interviewed and examined at MECs. After rigorous screening, 3242 women participants were finally included. The exclusion criteria are as follows: (1) under the age of 50 (*n* =  11004); (2) not participating in the detection of DXA (*n* = 1073); (3) answering ‘had regular periods in past 12 months (variable RHQ031)’ in Reproductive Health Questionnaire (*n* = 218); (4) data missing on related covariates (*n* = 2375). The NHANES website (https://www.cdc.gov/nchs/nhanes/) has comprehensive information on the survey's design, methodology, and statistics.

### Total BMD measurements and OP diagnosis

The BMD (gm/cm2) of the participants was measured by DXA when they were visiting an MEC. Briefly, the BMD measurements were performed with the Hologic QDR-4500A fan-beam densitometers (Hologic, Inc., Bedford, Massachusetts) for survey years 2007–2010. The femur and spine scans from 2007 to 2010 were analyzed with the Hologic software Discovery v12.4 and APEX v3.0, respectively. The Hologic APEX version 4.0 software was used to analyze both femur and spine BMD scans from 2013 to 2014. A previous study confirmed that there was no difference between the mean BMD values obtained by Hologic Discovery v12.4 and by APEX v 4.0 [32]. The BMD of the lumbar spine was defined as the mean BMD of the first lumbar vertebra to the fourth lumbar vertebra. The BMD of the neck of the left femur was generally taken as the femoral neck BMD, and the right hip was scanned if a left hip replacement or a metal object in the left leg was reported [33]. The exclusion criteria for the evaluation of DXA followed NHANES recommendations. Participants with fractures, receiving hip replacement or hip nail treatment, weighing over 300 pounds, with pregnancy (a positive urine pregnancy test result and/or self-reported pregnancy), or engaged in nuclear medicine research in the past 3 days were not recommended for DXA examination [34].

The diagnosis of OP should be made as per the guidelines of the World Health Organization (WHO). Presently, OP is confirmed when the BMD of a postmenopausal female interviewee is or lower than 2.5SD of the BMDs of healthy female adults [35]. In the present study, the BMDs in the lumbar spine and femoral neck were measured, and the latest BMD data updated by Xue et al. [36] were adopted as the reference.

### Serum Klotho concentration

The serum samples of the interviewees were collected, placed on dry ice and stored at -80℃ for subsequent analysis. Klotho concentrations in frozen NHANES 2007–2016 samples were determined by a commercially available Enzyme Linked Immunosorbent Assay (ELISA) kit produced by Immuno-Biological Laboratories (IBL) International, Japan [37]. All samples were analyzed in duplicate, and the average of the two concentrations was calculated as the result. One quality control sample with a low Klotho concentration and one quality control sample with a high Klotho concentration were also separately analyzed twice by ELISA. The analysis results were automatically transferred to the Laboratory Oracle Management system for evaluation by the area supervisor. Samples sharing more than 10% identical data were considered duplicates. If the value of the quality control sample was not within 2SD of the specified value, related data obtained were discarded and the sample analysis was repeated. The assay sensitivity was 6 pg/mL, and the final values of all samples exceeded this limit, so no interpolation was performed.

### Covariates

Information of age, gender and ethnicity was extracted from the demographic data.

Dietary data contained dietary calcium, which was assessed by 24-h dietary recall (24HR) interviews.

Body Mass Index (BMI) was obtained from examination data. Participants were divided into non-obesity (BMI < 25 kg/m2), overweight (25 kg/m2 ≤ BMI < 30 kg/m2) and obesity (BMI ≥ 30 kg/m2) according to the Centers for Disease Control and Prevention (CDC) standard.

Serum calcium, Klotho, creatinine and other serum testing data were extracted from laboratory data.

Participants did several questionnaires while coming in the MEC. Trained interviewers asked questions in a standardized protocol, and the answers were recorded in the file according to interviewees’ report.

More detailed information about BMD, Klotho and covariates is available at NHANES web (http://www.cdc.gov/nchs/nhanes/) or Supplementary Table 1.

### Statistical analysis

A descriptive analysis of the OP status and characteristics in participants was made in the present study. Continuous variables were expressed with mean and SD (Mean ± SD) or quartile [P_25_, P_75_], and compared by a T test or a Kruskal–Wallis test. Categorical variables were expressed with counts and percentages, and compared by a chi-square test. These statistical tests were conducted to examine if there were any differences in characteristics among participants in different bone states.

Klotho was regarded as a continuous variable. When evaluating the correlation between Klotho's logarithm and OP, multivariate binary logistic regression was used. The influence of Klotho on the incidence of OP was evaluated by a nomogram. Klotho was then divided into quartiles, and quartile 1 served as the reference group. A forest map was drawn to illustrate the effect of different subgroups on the occurrence of OP. Subgroup analyses of possible confounding covariates were performed with interaction terms. The restricted cubic spline (RCS) was applied to evaluate the risk of OP in participants with different Klotho levels. Missing data were removed from the analysis.

The *P* for trend was used to analyze the changing trend of OP morbidity with Klotho levels. We converted the variable of interest, Klotho, into a dummy variable according to the quartile. The dummy variable was taken as a new variable. Then, a regression model was constructed to explore the relationship between the dummy variable and outcome variable. Generally, the *P* for trend is the linear trend test result obtained by the regression model. The *P* for trend not only enhances the ability of models to detect risks, and but also detects whether there is a linear relationship between variables.

All analyses were performed using software package R-4.2.2 (http://www.R-project.org (The R Foundation)). *P* < 0.05 indicated statistical significance. Particularly, subjects with *P* < 0.1 in the single factor analysis were included in the multifactor analysis.

## Results

### Descriptive characteristics

A total of 871 postmenopausal women were included in the data analysis, and 89 (10.22%) of them had OP. The descriptive characteristics of the interviewees grouped by OP status are shown in Table 1. Compared to women without OP, those with OP were older and more lightweight, had higher serum phosphorus and Klotho concentrations and more pregnancies, and were more vulnerable to CKD. The risk of OP occurrence showed a strong correlation with the ethnicity.

**Table 1:** Characteristics of bone status in postmenopausal women

Variables	Non-OP (*n* = 796)	OP (*n* = 89)	*P-*value
Age (year)	61.9 ± 7.5	65.7 ± 7.5	< 0.001 **
Race			< 0.001 **
Mexican American	135(17.3)	11(12.36)	
Non-Hispanic black	155(19.8)	7(7.87)	
Non-Hispanic white	351(44.9)	42(47.19)	
Other Hispanic	92(11.8)	13(14.61)	
Other race^a^	49(6.3)	16(17.98)	
Dietary energy (kcal)	1651.8 ± 573.7	1679.5 ± 703.6	0.721
Dietary protein (gm)	65.4 ± 24.2	64.2 ± 26.2	0.677
Dietary sodium (mg)	2725.2 ± 1049.9	2647.4 ± 1044.9	0.507
Dietary potassium (mg)	2366.5 ± 880.7	2289.3 ± 951.1	0.467
Dietary calcium (mg)	814.5 ± 401.0	830.1 ± 494.2	0.774
Dietary phosphorus (mg)	1117.5 ± 401.1	1102.3 ± 482.7	0.776
BMI (kg/m2)	30.1 ± 6.5	25.4 ± 5.2	< 0.001 **
BMI^b^			< 0.001 **
Non obesity	170(21.7)	46(51.69)	
Overweight	364(46.5)	12(13.48)	
Obesity	248(31.7)	31(34.83)	
Serum total calcium (mmol/l)	2.4 ± 0.1	2.4 ± 0.1	0.490
Serum total phosphorus (mmol/l)	1.26 ± 0.17	1.30 ± 0.18	0.087 *
Log2.Klotho (pg/ml)^c^	9.70[9.40,10.00]	9.57[9.27,9.80]	0.003 **
Pregnancy times^d^	2.8 ± 1.9	3.2 ± 2.0	0.091 *
CKD			0.052 *
No	700(89.5)	73(82.02)	
Yes	82(10.5)	16(17.98)	
Smoking history			0.380
Never	421(53.8)	50(56.18)	
Former smoker	234(29.9)	21(23.60)	
Smoker	127(16.2)	18(20.22)	
Drinking history			0.584
Never	145(18.5)	21(23.60)	
Former drinker	170(21.7)	20(22.47)	
Mild drinker	257(32.9)	25(28.09)	
Moderate drinker	134(17.1)	12(13.48)	
Heavy drinker	76(9.7)	11(12.36)	
Hypertension			0.844
No	303(38.7)	36(40.45)	
Yes	479(61.3)	53(59.55)	
Diabetes^e^			0.830
No	481(61.5)	57(64.04)	
IGT	93(11.9)	11(12.36)	
Yes	208(26.6)	21(23.60)	
CVD			0.543
No	690(88.2)	76(85.39)	
Yes	92(11.8)	13(14.61)	
COPD			0.458
No	725(92.7)	80(89.89)	
Yes	57(7.3)	9(10.11)	

*BMI* Body mass index, *CKD* Chronic kidney disease, *CVD* Chronic vascular disease, *COPD* Chronic obstructive pulmonary disease

a.Other race including others not mentioned above, including multi-racial

b.Classify BMI according to CDC standard

c.Logarithm of plasma Klotho

d.Refers to the number of pregnancies over 28 weeks, including live births and stillbirths

e.Classification according to ADA-2013 guidelines

^*^P < 0.1

^*^*P < 0.05

### Multivariable models

Multivariate linear regression analysis showed that age, body shape and Klotho were independent risk factors for OP (Table 2). A higher concentration of Klotho contributes to a lower risk of OP. The morbidity of OP decreases by 0.568 for every doubling of the Klotho level (95%CI: 0.343–0.940, *P* = 0.027). Significant variables in multivariate analysis (*P* < 0.05) were used to construct logistic regression models and make forest plots.

**Table 2:** Logistic regression of bone status in postmenopausal women^a^

Variables	β	Odds Ratio(95% CI)	*P*-value
Age(year)	0.059	1.060(1.026–1.096)	< 0.001***
Race			
Mexican American	reference		
Non-Hispanic black	-0.723	0.485(0.165–1.341)	0.170
Non-Hispanic white	-0.006	0.994(0.476–2.213)	0.987
Other Hispanic	0.397	1.487(0.601–3.739)	0.390
Other race^b^	0.878	2.406(0.958–6.200)	0.063
BMI^c^			
Non obesity	reference		
Overweight	-0.828	0.437(0.255–0.740)	< 0.001***
Obesity	-2.122	0.120(0.057–0.236)	0.002 **
Serum total phosphorus(mmol/l)	-0.067	0.935(0.238–3.592)	0.923
Log2.Klotho(pg/ml)^d^	-0.565	0.568(0.343–0.940)^e^	0.027 *
Pregnancy times^f^	0.110	1.117(0.990–1.257)	0.069
CKD^g^			
No	reference		
Yes	0.438	1.550(0.765–3.025)	0.210

*BMI* Body mass index, *CKD* Chronic kidney disease

a.AIC:508.82

b.Other race including others not mentioned above, including multi-racial

c.Classify BMI according to CDC standard

d.Logarithm of plasma Klotho

e.The OR given is for each doubling of serum Klotho concentration

f.Refers to the number of pregnancies over 28 weeks, including live births and stillbirths

g.Classification according to ADA-2013 guidelines

.*P* < 0.1

^*^P < 0.05

^**^P < 0.01

^***^P < 0.001

### Development of a predictive model

**Fig. 1:**
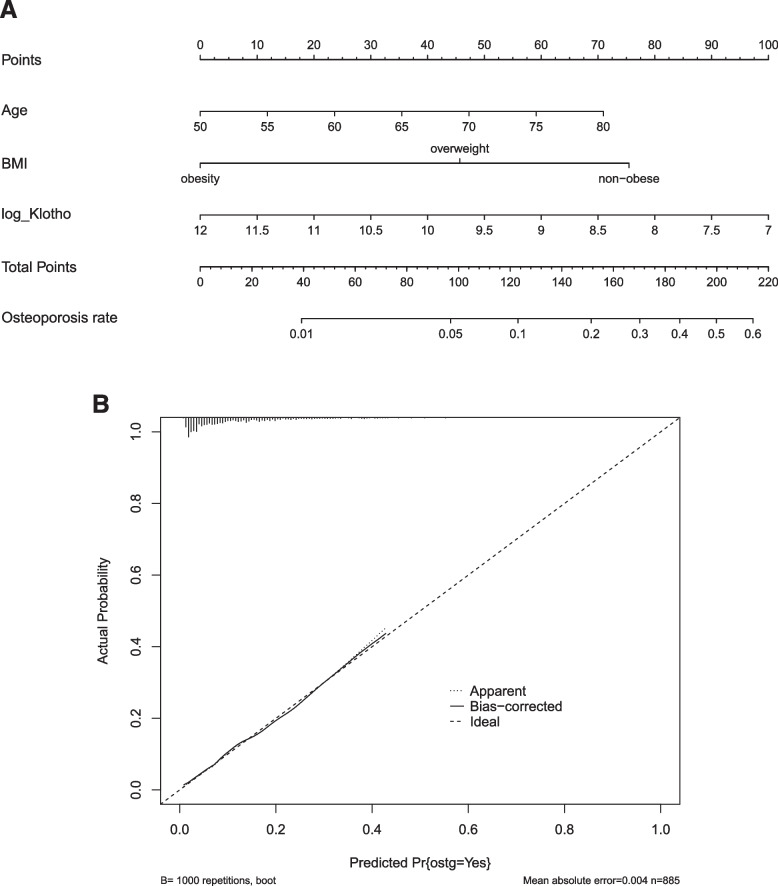
Nomogram of risk factors of osteoporosis and its correction curve. **A** plots the risk factors for osteoporosis. **B** is the correction curve of the model in Fig. 1A, where C = 0.765. Note: BMI: Body mass index, log_Klotho: The logarithm value of Klotho based on 2, Predicted Pr: Predicted probability

Firstly, we made use of three independent risk factors, namely, age, body shape and Klotho, to predict the risk of OP, and drew a nomogram based on the results of logistic regression (Fig. 1A). The nomogram has 6 lines in total, and Lines 2–4 indicate the three predictive factors. Each variable was assigned scores according to the scoring table (Line 1). The total score of each variable was calculated, and then a vertical line was drawn downward at the total score position (Line 5) to yield the probability of OP (Line 6). A higher total score indicates that OP is more possible to occur. A higher Klotho concentration leads to lower points and a lower total point, which means the probability of OP predicted by the nomogram is lower. Secondly, we evaluated the predictive ability of the nomogram using the calibration curves (Fig. 1B). The x-axis provides the predicted probability of OP, and the y-axis shows the incidence of OP in practice. The diagonal dotted line represents a perfect prediction, and the solid line exhibits the performance of the nomograph model constructed in the present study. Generally, the closer the solid line is to the dotted line, the better prediction performance the nomograph has. The model built in the present study was well calibrated since the C-index of the calibration curve is 0.765.

### Visualization of effective factors

**Fig. 2:**
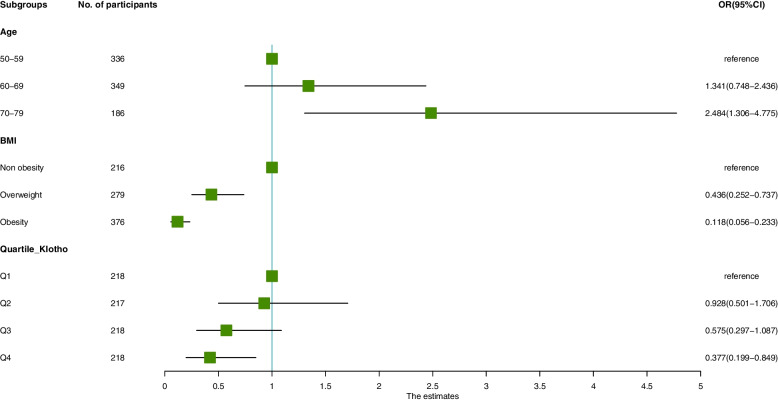
Forest map: OR value of osteoporosis. Figure shows the OR value of each variable

Participants were evenly divided into 4 groups based on the Klotho level (pg/ml), namely, Q1 (Klotho < 666.85), Q2 (666.85 ≤ Klotho < 822), Q3 (822 ≤ Klotho < 1004.65) and Q4 (Klotho ≥ 1004.65). We made a visual forest plot to describe the influence of effective factors in different subgroups (Fig. 2). When Klotho is considered as a categorical variable, the risk of OP is significantly increased in Q4 with the highest Klotho level, compared with that in Q1 (OR = 0.377, 95%CI: 0.199–0.849, *P* = 0.019). However, the OR of OP does not increase linearly as the Klotho level rises by one quartile.

### Trend for ORs

Table 3 shows the ORs of Klotho quartiles for OP in different adjusted models. In general, with the increase of the Klotho level, the risk of OP gradually decreases. The risk of OP in Q4 with the highest quartile (Q4) is about 1/3 of that in Q1. The trend test was made to examine whether this tendency was statistically significant. After adjusting the age, ethnicity, blood phosphorus, CKD and number of pregnancies in model 3, the change was found significant (*P* for trend = 0.047).

**Table 3:** Adjusted ORs(with 95%CI) for associations between Klotho and the prevalence of the OP

Adjusted model	Quartile of Klotho				*P* for trend^d^
	Q1	Q2	Q3	Q4	
Model 1^a^	reference	0.898 (0.484–1.652)	0.556 (0.287–1.053)	0.407 (0.192–0.820)*	0.166
Model 2^b^	reference	0.796 (0.435–1.443)	0.545 (0.285–1.015)	0.392 (0.189–0.774)**	0.123
Model 3^c^	reference	0.716 (0.396,1.280)	0.534 (0.283,0.981)*	0.341 (0.166,0.667)**	0.047

a.Model 1 is the full medel

b.Medel 2 is adjusted for race, serum total phosphorus and CKD

c.Medel 3 is adjusted for age, pregnancy times additionally by Model 2

d.P for trend based on variable containing median value for each quatile

^*^ *P* < 0.05 for logistic regression

. *P* < 0.1 for logistic regression

### Exploration of non-linear changes

**Fig. 3:**
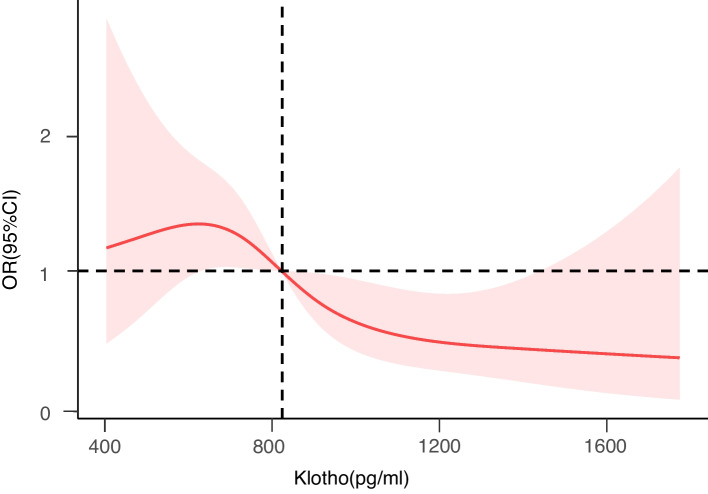
Restricted Cubic Spline for Model3. The unit of Klotho concentration is pg/ml. The concentration corresponding to the vertical dotted line is 824.09 pg/ml

We drew a RCS to describe the non-linear relationship between Klotho and OP on the basis of model 3, and built a visual prediction model (Fig. 3). It is evident that Klotho does not affect OP continuously. The curve is an inverted U shape. The incidence of OP maintains relatively stable at first, but after the Klotho level reaches 824.09 pg/ml, it decreases rapidly (non-linear *P* = 0.0289). However, when the Klotho concentration is extremely low or high, the result of the prediction performance is not accurate.

## Discussion

OP is a major health problem for postmenopausal women. In order to evaluate the risk of OP and prevent OP, an increasing number of evaluation tools have emerged and been developed. The FRAX released by the WHO Cooperation Center in Sheffield, UK in 2008, is the most extensively used tool to assess bone health. However, further research on OP has identified more and more risk factors.

In the present study, the results indicated that Klotho was independently associated with OP in postmenopausal women (OR: 0.568(0.343–0.940),* P* = 0.023). Compared with that in the group with the lowest Klotho level, the risk of OP in the group with the highest Klotho level was reduced by 65.9%. The RCS results showed that when the serum Klotho level was higher than 824.09 pg/ml, the risk of OP decreased sharply. Subgroup analysis suggested that after all variables were adjusted, the *P* for trend was significant, indicating that the OP incidence in participants drops more significantly with the increase in BMD. Compared with previous studies, the present study confirms that age and body type are independent risk factors of OP.

The present research has reached a conclusion similar to that of the studies by Kužmová Z et al. [27] and Baldan A et al. [28]. That is, Klotho is a protein that protects bones. A higher Klotho level is associated with a lower incidence of OP, which means that a high Klotho concentration can enhance the stability of bone trabecular structures and reduce the incidence of osteoporotic fractures. A clinical research found that serum Klotho levels in patients with a low BMD was statistically significant lower than that in patients with a normal BMD [38]. However, the results of the present study are inconsistent with those reported by De-la–O A et al. [29] Considering that they studied a group of men and women at a specific age,, a sedentary lifestyle may also be one factor influencing the occurrence of OP. In addition, Neyra JA et al. [39] claimed that Klotho inhibited the expression of bone FGF-23 in healthy people, while in CKD people, low Klotho levels promoted the production of bone FGF-23. Therefore, Klotho may have different functions in different health conditions.

In the past few years, the discovery of the FGF-23/Klotho axis and its impact on phosphorus and calcium metabolism have changed our understanding of mineral metabolism regulation. FGF-23 is a key hormone regulator of calcium metabolism, phosphate levels and vitamin D levels, having the physiological functions of maintaining bone health and the systemic mineral balance [20, 40]. FGF-23 is mainly secreted by osteoblasts in bone tissue and responds to the increased phosphate and calcium loading, calcitriol, parathyroid hormone (PTH) and bone remodeling. As a growth factor synthesized in bone, FGF-23 inhibits the activation of vitamin D in the kidney and induces phosphate excretion through proximal tubule epithelial cells [41]. Klotho is known as a membrane protein that responds to the effects of FGF-23 on the kidneys [42]. Most recently, it was reported that the FGF23-Klotho axis is associated with an increasing incidence of fractures [43] and the change in Klotho levels was closely related to BMD in postmenopausal women [37].

According to a previous study, Klotho proteins inhibit the expression of 25-(OH)D_3_ 1α-hydroxylase in kidney cells in vitro [44], a key enzyme for VitD_3_ activation. It is well established that the activation of the FGF23/α-Klotho signal reduces the serum VitD_3_ level by hindering Cyp27B1-mediated formation and stimulating Cyp24A1-mediated catabolism of 1,25-(OH)_2_D [20, 23, 45, 46]. It is acknowledged that 1,25-(OH)_2_D, a steroidal hormone, acts mainly as a ligand of the widely-distributed nuclear vitamin D receptor (VDR). Vitamin D compounds reduce the ratio of RANKL/OPG and mitigates osteoclast differentiation by acting on VDRs, which are preferentially expressed in osteoblasts and osteocytes. However, mineral-regulating hormones such as PTH and FGF-23 play a complex role in the VDR-regulated expression of RANKL and OPG in osteoblast lineage cells [47]. The study of Chai Y et al. [48] shows that Astragalus membranaceus up-regulates the expression of Klotho, VDR, and Cyp27B1, down-regulates the expression of FGF23 and CYP24A1, and raises the calcium and phosphorus levels in mice, thus improving the femoral BMD and bone microstructure.

Klotho-deficient mice shows high expression of FGF-23 [49]. It is generally acknowledged that FGF23 reduces serum phosphate levels by inhibiting 1,25-(OH)_2_D synthesis, suppressing intestinal phosphate absorption, and down-regulating the expression of transporters NPT2a and NPT2c, curbing phosphate reabsorption in proximal tubules [50]. When bone remodeling occurs, osteoblasts secrete FGF-23, which binds to FGFR1-Klotho in proximal and distal tubules of kidneys, directly activating ERK 1/2 and SGK-1 signaling cascades. Consequently, the expression of the Na/H exchange regulatory cofactor (NHERF)-1 is down-regulated, which is localized to the brush border membrane of proximal tubular cells, a major site of phosphate reabsorption [46]. Down-regulation of NTP2A weakens phosphate reabsorption activities in proximal tubules and promotes urinary phosphate excretion [16, 51]. The parathyroid gland also expresses FGFRs, and their combination with the ligand inhibits the secretion of PTH. Meanwhile, FGF-23 suppresses the activation of vitamin D in the kidney, thus stimulating the synthesis of PTH [52]. Both of these pathways encourage bone formation and alleviate bone degradation. Klotho serves as a mediator that realizes FGF-23 binding to its receptor and increases the affinity between FGFRs and FGF-23 [21]. Klotho deficiency will impair phosphorus reabsorption in the kidney, leading to secondary hyperparathyroidism and ultimately bone formation disorders.

Moreover, WNT1 secreted by bone cells is directly involved in bone formation [53, 54]. It has been confirmed that the extracellular domain of Klotho binds to multiple Wnt ligands, rendering them incapable of activating Wnt signaling [55, 56]. In the process, FGF23 inhibits Wnt signaling through the increase of Dkk1 levels induced by soluble Klotho in bone [57]. Another study indicates a direct relationship among FGF23, reduced Wnt activity, and bone demineralization [58]. What's more, with the assistance of Klotho, FGF23 can inhibit Wnt signaling and osteogenesis by enhancing the Dkk1 expression. Hence, Wnt activity is increased in Klotho knockout mice [59].

In fact, NF-κB plays a critical role in the connection between cardiovascular disease and OP. A mice study has confirmed that the inhibition of NF-κB leads to osteoblast differentiation and bone enhancement [60]. It is verified that Klotho stimulates osteoclastogenesis through the activation of the NF-κB signaling pathway [61, 62].

To our best knowledge, the present study is the first to use a national sample to explore the relationship between OP and Klotho. The NHANES is conducted based on a large, multi-ethnic, representative population from the United States, so it enhances the statistical effectiveness of the present research and renders the results more convincing. In addition, we adjusted all the potential confounding factors when exploring the relationship between OP and Klotho levels, thus improving the accuracy of the research results. However, the present research also has some limitations. Firstly, we cannot use the VitD, PTH, physical activity and dietary supplement data collected by the NHANES for analysis due to the following reasons. The VitD collected by the NHANES database is not the biologically active VitD3 but VitD2. The database does not contains any PTH survey data in the years studied. Besides, there are a large number of missing values on physical activity and dietary supplements. Unfortunately, the BMD data from 2011–2012 cannot be read by the NHANES. Secondly, the NHANES is a cross-sectional study, so it is difficult to determine the relationships between temporality and causes. Moreover, due to no response to the NHANES survey and the missing values of some variables, biases might arise from the exclusion of participants from the analysis. Thus, longitudinal studies are required to provide serial serum Klotho measurements to address temporality. Thirdly, given the differences among different ethnic groups, the accuracy of the modeling results entails additional evaluation before applying the results to ethnic groups in other countries or regions.

In conclusion, serum Klotho levels are negatively correlated with the prevalence of OP in a nationally representative sample. A higher serum Klotho level is related to a lower incidence of OP. The Klotho level is a factor influencing the occurrence of OP. The findings of the present study can provide guidance for research on diagnosis and risk assessment of OP.

### Supplementary Information

**Additional file 1:** **Supplementary Table 1.** Variable Table.

## Data Availability

The datasets generated during or analysed during the current study are available in the NHANES repository (http://www.cdc.gov/nchs/nhanes/).

## Abbreviations

OP: Osteoporosis
DXA: Dual-energy X-ray absorptiometry
BMD: Bone mineral density
SD: Standard deviation
FRAX: Frame risk assessment tool
*KL*: Klotho gene
CVD: Cardiovascular disease
TBS: Trabecular bone score
NHANES: National health and nutrition examination survey
MECs: Mobile examination centers
CKD: Chronic kidney disease
