# Correction: Klotho reduces the risk of osteoporosis in postmenopausal women: a cross-sectional study of the National Health and Nutrition Examination Survey (NHANES)

**DOI:** 10.1186/s12902-024-01628-y

**Published:** 2024-06-20

**Authors:** Jialin Jiang, Qinyu Liu, Yaqian Mao, Nengyin Wang, Wei Lin, Liantao Li, Jixing Liang, Gang Chen, Huibin Huang, Junping Wen

**Affiliations:** 1 Shengli Clinical Medical College of Fujian Medical University, Fuzhou, China; 2 Department of Endocrinology, Fujian Provincial Hospital, Fuzhou, China; 3 Department of Internal Medicine, Fujian Provincial Hospital Jinshan Branch, Fuzhou, China; 4 Fujian Provincial Key Laboratory of Medical Analysis, Fujian Academy of Medical, Fuzhou, China

**Correction:** ***BMC Endocr Disord*** **23, 151 (2023)**

10.1186/s12902-023-01380-9

Following publication of the original article [1], the authors reported an affiliation for Junping Wen was missed at the time of publication.

The affiliation of Junping Wen in the published article is:

Department of Endocrinology, Fujian Provincial Hospital, Fuzhou, China.

The correction affiliations of Junping Wen should be:

Shengli Clinical Medical College of Fujian Medical University, Fuzhou, China.

Department of Endocrinology, Fujian Provincial Hospital, Fuzhou, China.
