# Supplementary material for: Klotho reduces the risk of osteoporosis in postmenopausal women: a cross-sectional study of the National Health and Nutrition Examination Survey (NHANES)
Source: BMC Endocr Disord. 2023 Jul 14;23:151. doi: 10.1186/s12902-023-01380-9 (PMC10347835; doi:10.1186/s12902-023-01380-9)
Supplement: Supplementary file 1 — Additional file 1: Supplementary Table 1. Variable Table. [file 12902_2023_1380_MOESM1_ESM.doc]

**Supplementary Table 1: Variable Table**

| Items | English Text | Document file | Variable | Description |
| --- | --- | --- | --- | --- |
| Demographics | Gender | demo | riagendr |  |
|  | Age | demo | ridageyr |  |
|  | Race | demo | ridreth1 | Including Mexican American, non-Hispanic black, non-Hispanic white, other Hispanic and other races. |
| Dietary | Dietary calcium intake | dr1tot, dr2tot | dr1tcalc, dr2tcalc | All participants had two dietary assessments, and we calculated the arithmetic mean of the specific nutrient on two days as the final nutrition intake result. |
| [Examination](https://wwwn.cdc.gov/nchs/nhanes/search/datapage.aspx?Component=Examination&CycleBeginYear=2015) | Height | bmx | bmxht |  |
|  | Weight | bmx | bmxwt |  |
|  | BMI | bmx | bmxbmi |  |
|  | Femoral neck BMD | dxxfem | dxxnkbmd |  |
|  | Lumbar Spine BMD | dxx | dxxlsbmd |  |
| [Laboratory](https://wwwn.cdc.gov/nchs/nhanes/search/datapage.aspx?Component=Laboratory&CycleBeginYear=2015) | Total calcium | biopro | lbdscasi |  |
|  | Creatinine | biopro | lbdscrsi |  |
|  | Klotho | sskl | sskloth |  |
| Questionnaire | Had regular periods in past 12 months | rhq | rhq031 |  |
|  | Reason not having regular periods | rhq | rhd042, rhd043 |  |
|  | How many vaginal deliveries? | rhq | rhq166 | The pregnancy times was the sum of vaginal delivery and cesarean section, including live births and stillbirths. |
|  | How many cesarean deliveries? | rhq | rhq169 |  |
| Secondary calculation | Osteoporosis | diag_osteoporosis |  |  |
|  | eGFR | dex_eGFR |  | The eGFR was calculated by MDRD-2007 simple formula: eGFR(ml/(min*1.73m2))=186*(Scr)^-1.154*(age)^-0.203*0.742. The grading of chronic kidney disease (CKD) referred to the Kidney Disease: Improving Global Outcomes (KDIGO) standard and was divided into five grades according to eGFR |
|  | CKD | diag_CKD |  | CKD was diagnosed if eGFR was less than 60ml/(min*1.73m2). |
|  | Smoke | diag_smoke |  | Smoking history was divided into three categories by uses of cigarettes. |
|  | Alcohol use | diag_alcohol.user |  | There were five classes of alcohol usage according to the methods of Rattan et al.(Rattan P, 2022) |
|  |  | diag_Hypertension |  | Hypertension was diagnosed comprehensively according to past history, drug usage and blood pressure measurement referred to diagnosis of JNC 7(systolic blood pressure ≥140 mmHg and/or diastolic blood pressure ≥90 mmHg). |
|  |  | diag_DM |  | Impaired fasting glucose(IGT)/DM status was based on current ADA 2013 criteria, combined with self reports and drug use. |
|  |  | diag_CVD |  | CVD results included self-reported physician diagnoses of coronary heart disease (CHD), congestive heart failure (CHF), angina, heart attack, or stroke. |
|  |  | diag_COPD |  | COPD was diagnosed by spirometry, emphysema diagnosis, and medication record. |
